# Dynamic Trajectory of Long-Term Cognitive Improvement Up to 10 Years in Young Community-Dwelling Stroke Survivors: A Cohort Study

**DOI:** 10.3389/fneur.2019.00097

**Published:** 2019-02-12

**Authors:** Eva Elgh, Xiaolei Hu

**Affiliations:** ^1^Department of Psychology, Umeå University, Umeå, Sweden; ^2^Department of Community Medicine and Rehabilitation, Umeå University, Umeå, Sweden

**Keywords:** cognition, cognitive improvement, cognitive impairment, stroke, young adults, longitudinal study

## Abstract

**Background and objective:** The trajectories of long-term and domain-specific cognitive alterations over a decade after stroke are largely unknown. This study aims to investigate the dynamic alterations of domain-specific cognitive performance among young stroke survivors over 10 years after their first stroke.

**Methods:** A prospective cohort study was carried out on 38 young stroke survivors (aged 18–65 at stroke onset) living in the community at 10 years after their first stroke. The cognitive outcomes were assessed repeatedly at 1 week, 7 months, and 10 years after their first stroke on the sub-domains: process speed *(Symbol search and Coding from WAIS, TMT-A)*, visual attention *(Bells test)*, visuospatial function *(Block design from WAIS, RCFT)*, executive function (*TMT-B, verbal fluency*), verbal function *[Letter fluency (FAS) from D-KEFS and CD]*, working memory (*Digit Span from WAIS*), immediate memory (*RCFT and CD*), and delayed memory (*RCFT and CD*). Global cognition was evaluated with Mini mental state examination at the two later time-points.

**Results:** We found a delayed significant improvement of working memory with total recovery 10 years after participants' stroke. Visuospatial function recovered already at 7 months and remained stable at 10-year follow-up. Process speed demonstrated a significant decrease at 10 years compared to 7 months after stroke onset, a decrease which could be compensated by enhancements of other cognitive domains. No further deterioration was found in verbal function, immediate-, and delayed memory, and executive function during 10-year follow-up. Global cognition improved by on average two points between 7 months and 10 years. Education level and fatigue showed low to moderate positive correlations with cognitive improvements.

**Conclusions:** The concordance of cognitive improvements between domain-specific and global cognitions strongly suggest that some young stroke survivors do improve their cognitive outcome over a 10-year period following their first stroke. This finding fills a gap of knowledge with respect to the dynamic trajectory of post-stroke cognition, with important implications in clinical practice.

## Introduction

Stroke is the second most common cause of acquired cognitive impairment and is strongly associated with increased disability, dependency, institutionalization, and mortality (1). Thrombolysis, improvements on rehabilitation, and secondary stroke prevention during the latest two decades have significantly reduced stroke mortality, which prolongs life expectancy with extended disability-adjusted life years (DALYs) among stroke survivors, particularly younger ones (2). While many young stroke survivors may live several decades after stroke onset, knowledge regarding the long-term outcomes and longitudinal care after stroke remain poorly investigated. Cognitive impairment as one of the most “invisible disabilities” has often been overlooked among stroke survivors during follow-up, despite its significant impact on their lives.

Currently, the few studies regarding the longitudinal effect of stroke on cognition have shown mixed results, demonstrating either a trend toward deterioration (3–5), stability (6, 7), or improvements (8–11) over time. Notably, these previous studies are based mostly upon simple global cognitive assessment instead of comprehensive assessment with a neuropsychological test battery (12). Two high-quality studies with sufficient sample size have shown cognitive decline at 6 and 10 years after stroke, but assessed only cognition at one single time-point (3, 4). These studies failed to demonstrate dynamic cognitive alterations after stroke, despite acute and late post-stroke cognitive declines often being reported (12). Therefore, trajectories of long-term and domain-specific cognitive alterations among stroke survivors over a decade are largely missing.

The aim of the current study was to investigate dynamic alterations of the domain-specific and global cognitive performances among young stroke survivors over 10 years after their first stroke.

## Materials and Methods

### Study Design

This study was a single-center prospective cohort study of stroke survivors with 10-year follow-up. It was carried out at the Department of Neurological Rehabilitation, University Hospital of Northern Sweden. Ethical approval was obtained from the regional Ethical Review Board in Umeå, Sweden, D-nr 2015/144-31.

### Recruitment and Participants

All young patients (>18 years to 65 years) who had suffered a first-ever stroke between January 2004 and December 2007 with neuropsychological assessment within the first year after stroke, at Stroke Centrum, University Hospital of Northern Sweden were contacted and provided informed and written consent via letter and telephone for recruitment to the study. Exclusion criteria were severe dementia, severe aphasia, and severe comorbidity/non-community-dwelling, recurrence of stroke/TIA and other physical and/or psychiatric disease after first-ever stroke.

**Figure 1:**
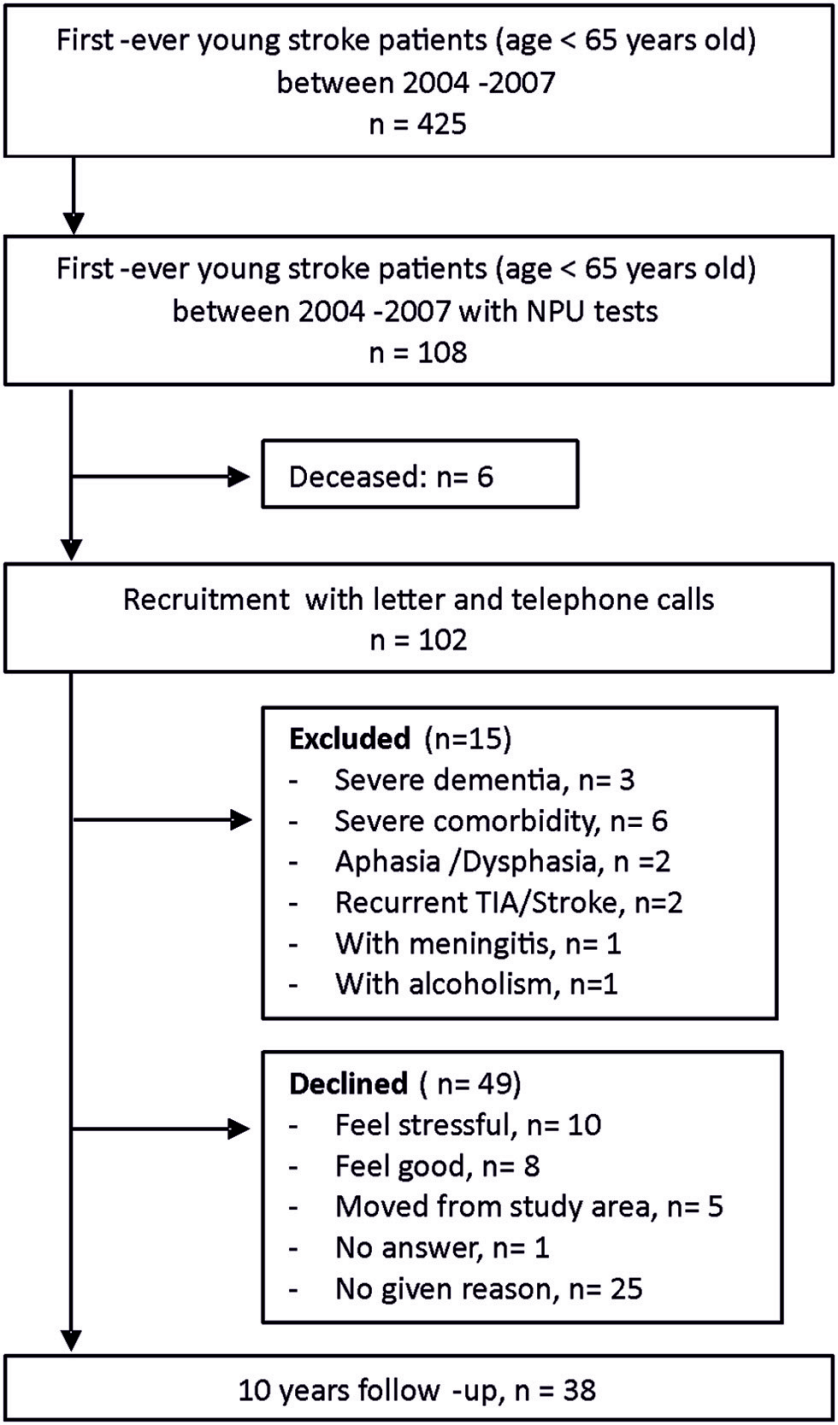
Flow diagram of inclusion process.

There was a total of 425 first-ever young stroke patients in the Riksstroke registry and medical journals between January 1st, 2004 and December 31st, 2007 within the catchment area. The Riksstroke registry has a proximately cover-rate of 86%. Neuropsychological assessments (NPAs) were performed for 108 of the young stroke patients within 1 year after their stroke. The reason for the initial NPA was unknown to us but possibly due to patients' capability to be assessed and access to a psychologist at that time. NPAs were often carried out within 1 week after stroke debut. Some of the participants were assessed again at approximately seven (IQR 2–10) months after stroke. After a thorough recruitment process, 38 stroke survivors participated in the 10-year follow-up with written consent, while 49 declined due to various reasons (Figure 1). All participants were native Swedish citizens.

### Cognitive Function Assessments

NPAs at 10-year follow-up were administrated between 2015 and 2016. The follow up assessments were completed by four assessors who were blinded to the previous assessments. The entire test battery took approximately 2–3 h, with an extra 30-min break with refreshments in the middle. To ensure comparability, the selection of tests at follow-up was based on the tests that had been used at the first assessment within 1 year after stroke. Notably, Wechsler Adult Intelligence Scale (WAIS)-R and WAIS-III was replaced by WAIS-IV at 10-year follow-up due to practical reasons. Previous validation studies have shown that WAIS-IV has the same construction as WAIS-III/R (13), with very high correlation between subscales (*r* = 0.82–94) (14). Swedish norms for WAIS-IV were used as control (14). In addition, Mini mental state examination (MMSE) was carried out immediately prior to NPAs.

The following cognitive domains were examined: process speed *(Symbol search and Coding from WAIS, TMT-A)*, visual attention *(Bells test)*, visuospatial function *(Block design from WAIS, RCFT)*, executive function (*TMT-B, verbal fluency*), verbal function *(Letter fluency (FAS) from D-KEFS and CD)*, working memory (*Digit Span from WAIS*), immediate memory (*RCFT and CD*), and delayed memory (*RCFT and CD*) (Table 2).

### Other Outcome Measurements

One month prior to the scheduled appointment for NPA, the participants received various questionnaires to complete at home and return on the NPA occasion. Depression, anxiety, and fatigue were assessed with the Beck Depression Inventory-II (BDI-II), Beck Anxiety Inventory (BAI), and Fatigue assessment scale (FAS). Baseline data were collected from the Riksstroke registry and medical records. Participants also provided information regarding their education level, weight, height, and employment status.

### Data Presentation and Statistical Analysis

Demographic characteristics are presented as mean ± SD, number with/without number of cases (%) or median [25–75% interquartile ranges (IQR)] as appropriate. Baseline characteristics in patients were compared using a Student *t*-test, Fisher's exact test, or Chi-square test when appropriate.

Data from NPA are presented in the medium raw scores with 25–75% IQR because of the limited and varying number of participants at the early stage. The NPAs at the early stage were presented at two time-points (i.e., 1 week and 7 months, with some missing values). No adjustment was made on missing values. Numbers of raw scores on each assessment /time-point are clearly presented in Table 2. A repeated measures ANOVA (Friedman test) or non-repeated measures ANOVA (Kruskal-Wallis test) was chosen for non-parametric multiple comparisons between three time-points, then a Wilcoxon matched-pairs signed rank test was used between two time-points. Each *p*-value was adjusted to account for multiple comparisons using Dunn's multiple comparisons test. A Spearman correlation test was used to obtain the analysis correlation coefficient. Statistical analyses were performed using GraphPad Prism software version 6.0., with a *P* < 0.05 being considered significant.

## Results

### Basic Demographic and Clinical Characteristics

Baseline characteristics of study participants, stroke survivors who declined participation and all first-ever young stroke patients within the catchment area under the same period are presented in Table 1. There were no significant differences with respect to any characteristic between participants and decliners as well as between first-ever stroke survivors with and without NPA at the early stage. However, there were significantly less ischemic strokes but more hemorrhagic strokes among patients with NPAs (*P* < 0.01).

**Table 1:** Demographic and clinical characteristics of all first-ever young stroke survivors within the catchment area between 2004 and 2007 and all participants at 10-years follow-up.

**Characteristics**	**First-ever young stroke survivor with NPA**				**First-ever young stroke survivor**	
	**Recruitment 10 years follow-up**			**All with NPA (*n* = 108)**	**All without NPA (*n* = 317)**	***p*-value**
	**Declined (*n* = 49)**	**Included (*n* = 38)**	***p*-value**			
**PATIENT CHARACTERISTICS AT STROKE ONSET**						
Mean age ± *SD*	54.9 ± 7.9	53.9 ± 9.1	0.58	54.7 ± 8.6	55.8 ± 8.4	0.18
Men/Women	33/16	19/19	0.13	70/38	207/110	>0.99
Civil status (Live alone/live with somebody/unknown)	8/39/2	7/30/1	0.91	22/82/3^a^	85/219/13	0.33
**STROKE CHARACTERISTICS AT STROKE ONSET**						
Ischemia (%)	35 (71%)	30 (79%)	0.47	81 (75%)	191 (86%)^b^	0.007^*^
Hemorrhage (%)	10 (20%)	6 (16%)	0.78	18 (17%)	24 (13%)^b^	0.009^*^
Unknown (%)	4 (8%)	2 (5%)	0.69	9 (8%)	4 (1%)^b^	0.001^*^
**RISK FACTORS AT STROKE ONSET**						
Atrial fibrillation (%)	2 (4%)	4 (11%)	0.40	6 (6%) ^a^	19 (6%)	>0.99
Hypertension (%)	19 (39%)	11 (29%)	0.37	36 (34%) ^a^	117 (37%)	0.56
Diabetes mellitus (%)	6 (12%)	4 (11%)	>0.99	12 (11%) ^a^	39 (12%)	0.86
Smoking (%)	16 (33%)	9 (24%)	0.47	30 (28%) ^a^	72 (22%)	0.30
**CHARACTERISTICS AT 10 YEARS-FOLLOW-UP**						
		**Total**		**Female**	**Male**	
Age (mean ± *SD*)		63.8 ± 10.6		61.8 ± 9.9	65.7 ± 11.3	0.14
Years between follow-up and stroke onset (mean ± *SD*)		10.5 ± 0.9		10.6 ± 0.9	10.4 ± 0.8	0.40
Median of mRS (25–75% percentile)		1 (0–2)^a^		1 (0–2)	0 (0–1.3)	0.21
Median of BMI (25–75% percentile)		26 (22–28)^c^		28 (23–30)	26 (22–27)	0.08
Median of BAI (25–75% percentile)		6 (2–11)		10 (3–17)	4 (1–7)	0.09
Median of BDI-II (25–75% percentile)		9 (4–14)		10 (6–16)	7 (2–11)	0.15
**EDUCATION (NUMBER OF CASES %)**						
9 years		7 (19%)		3 (16%)	4 (22%)	0.69
12 years		12 (32%)		8 (42%)	4 (22%)	0.30
>12 years		17 (46%)		8 (42%)	9 (50%)	0.75
**FULLTIME EMPLOYMENT (NUMBER OF CASES %)**						
Before stroke		28 (76%)^a^		14 (74%)	14 (77%)	>0.99
2 years after stroke		11 (32%)^d^		5 (29%)	6 (35%)	>0.99
10 years after stroke		4 (11%)^a^		1 (5%)	3 (17%)	0.34

* *P < 0.05 was considered as significance*.

a *Missing 1 patient in the group*.

b *30% missing value*.

c *24% missing value*.

d *10% missing value. ns, no significant difference; NPA, neuropsychological assessment*.

At 10-year follow-up (Table 1), the mean age of participants was 63.8 (*SD* 10.6) years. All participants were living in the community. Most participants (*n* = 34, 34/37, 92%) had no or only slight disability (mRS = 0–2), the remaining three participants had moderate disability (mRS = 3). More than two thirds of participants (*n* = 21, 21/29) were overweight (BMI >25). Approximately one third of participants (*n* = 15, 15/36) had anxiety problems (BAI >8), four participants (4/36, 11%) had moderate anxiety and two participants (2/36, 5%) suffered severe anxiety (BAI >25). Eleven participants (11/38, 29%) were depressed (BDI-II >13), with three having moderate (3/38, 8%) and one severe (1/38, 3%) depression (BDI-II >28). Almost half of the participants (*n* = 17, 17/37, 46%) had received education for more than 12 years. Four participants were still working full time (4/37, 10%); and six participants were working part time at 10-year follow-up. No significant difference with respect to basic characteristics was observed between men and women at 10-year follow-up (Table 1).

### Improved Working Memory Over 10-Year After Stroke

**Figure 2:**
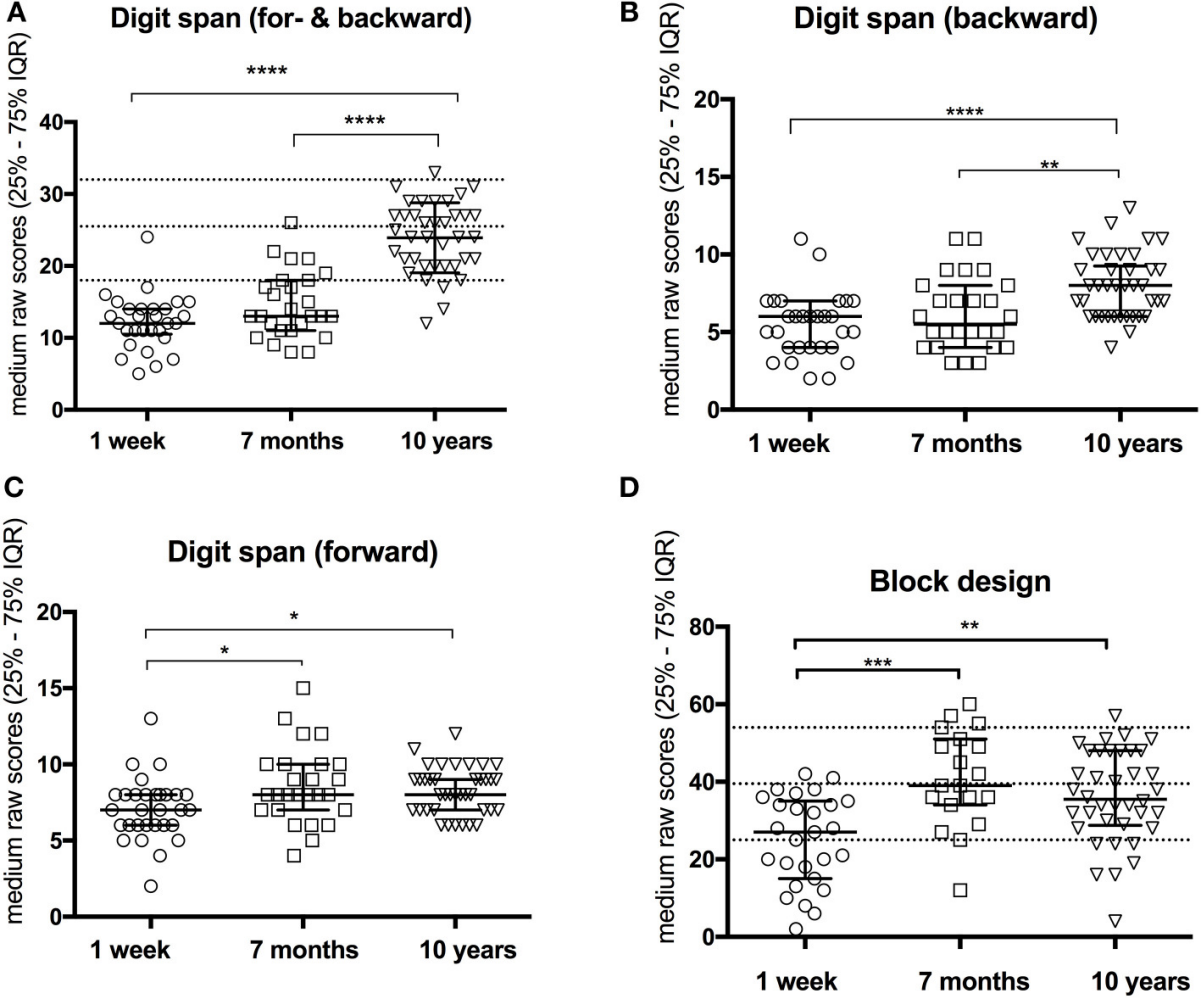
Improvement of working memory **(A–C)** and visuospatial function **(D)** over 10-year follow-up. Dotted lines in **(A,D)** indicate respective normative data (mean ± *SD*). *P < 0.05; **P < 0.01; ***P < 0.001; ****P < 0.0001.

Compared to results at 1-week post-stroke, no significant enhancements on working memory were observed at 7 months after stroke onset as assessed by WAIS-Digit span (14). However, significant improvements were demonstrated at 10-year follow-up in Digit span total score (10.72, 95%CI— 9.24 to 12.21, *P* < 0.0001) (Figure 2A and Table 2) and in Digit span backward alone (2.14, 95%CI— 1.39 to 2.89, *P* < 0.0001) (Figure 2B and Table 2) compared to 1 week after stroke onset as well as 7-month follow-up (9.42, 95%CI— 7.44 to 11.41, *P* < 0.0001, respectively 1.85, 95%CI— 0.89 to 2.8, *p* = 0.006). The digit span total score among participants reached a similar level as no-stroke individuals at age 55–64 years (14). Digit span forward showed a significant improvement already at 7-month follow-up (sub-acute phase) and remained stable at 10-year follow-up (Figure 2C and Table 2).

**Table 2:** Neuropsychological assessment scores from 1 week up to 10 years after stroke onset.

**Cognitive domain**	**Assessments**	**Time after stroke onset**						
		**1 week**		**7 months**		**10 years**		***p*-value**
		**Raw score**	***N***	**Raw score**	***N***	**Raw score**	***N***	
Global cognition	MMSE			27 (25–29)	17	29 (27–30)	38	0.01^*^
Processing speed	WAIS-Symbol searching	27 (23–37)	3	28 (20.5–35)^a^	21	23 (16.8–27)^b^	38	0.03^*^(a: b)
	WAIS-Coding	43 (31–46)^c^	23	57 (42–69)^d^	23	49 (35.5–56.5)	37	0.007^*^(c: d)
	TMT–A	46 (35–56)	27	40 (26–65)	11	37 (29.5–50)	37	0.27
Visual attention	Bells test	34 (30.5–35)	29	34 (32.5–34.5)	21	33.5 (31.8–35)	38	0.87
Verbal fluency	D-KEFS-FAS	35 (25.3–41.8)	24	38 (27.8–59.8)	8	34 (25.8–48)	38	0.65
	CD	See below						
Immediate memory	RCFT-IM	14 (10.3–17.8)	4	14 (10.6–21.8)	14	12 (7.8–16)	34	0.32
	CD-weighted score	230	1	138 (29–196)	19	127.5 (99.5–181.5)	38	0.47
Delayed memory	RCFT-delayed recall	12.5 (8.9–19.1)	4	14.3 (10.8–20.6)	14	10.5 (6.6–16)	36	0.14
	RCFT-recognition	19 (15.8–20.8)	4	20 (19–20)	13	19 (18.3–21)	36	0.68
	CD-retention	57	1	71 (50–100)	19	70 (56–86.5)	38	0.80
	CD-recognition	10	1	10 (9–10)	19	10 (9–10)	38	0.39
Executive function	TMT-B	101 (67–134)	27	72 (54–126)	11	81 (62–116)	37	0.17
	D-KEFS-FAS	See above						
Visuospatial function	WAIS-Block design	27 (15–35)^e^	27	39 (34–51)^f^	19	35.5(28.8–48)^g^	38	0.0003^*^(e: f)
								0.004^*^(e: g)
	RCFT-copy	31 (29.3–33.9)	4	31 (29–34.3)	14	28.5 (24.7–31.8)	36	0.11
Working memory	WAIS-Digit span (F)	7 (6–8)^h^	30	8 (7–10)^i^	26	8 (7–9)^j^	38	0.03^*^(h: i)
								0.02^*^(h: j)
	WAIS-Digit span (F + B)	13 (11–14)^k^	29	13 (11.75–18)^l^	26	24 (20–27)^m^	38	<0.001^*^(k: m)
								<0.001^*^(l:m)
	WAIS-Digit span (B)	6 (4–7)^n^	29	5.5 (4–8)^o^	26	8 (6–9.25)^p^	38	<0.001^*^(n: p)
								0.006^*^(o:p)

*Data presented as median (25–75% percentile)*.

* *P < 0.05 was considered as significance. ns, no significance; Nr, number of participants; WAIS-IV, Wechsler Adult Intelligence Scale-IV; RCFT, Rey Complex Figure Test and recognition trial; CD, Claeson-Dahl; FAS: a part of the test battery Delis-Kaplan Executive Function System (D-KEFS), named letter fluency. F, Forward; B, backward; MMSE, Mini Mental Scale Examination; TMT, Trail making test; IM, Immediate Memory*.

Immediate memory assessed by RCFT (15) and CD (16) showed no significant changes between 7-month and 10-year follow-ups (Table 2). Similar to immediate memory, delayed memory remained at the same levels when RCFT- delayed recall and recognition as well as CD- retention and recognition were examined at 7 months and 10 years after first-ever stroke (Table 2). Notably, only a few participants (*n* = 1 or 4) were tested in the memory domain at 1 week after stroke onset (Table 2).

### Improved Visuospatial Function Already at Subacute Phase (7 Months After Stroke)

The visuospatial function, assessed with WAIS Block design, showed a significant improvement (14.36, 95%CI— 10.78 to 17.95, *p* = 0.0002) already at 7 months after stroke onset compared to the results at 1-week post-stroke. The improvements remained at 10-year follow-up (9.96, 95%CI— 6.46 to 13.47, *p* = 0.003) (Figure 2D and Table 2) and reached the normative level compared to non-stroke peers (14). However, there were no significant changes in RCFT-copy test (Table 2).

### Stable Attention, Verbal Fluency, and Executive Function During 10-Year Follow-Up

Attention examined with Bells test (17) showed similar results from 1 week, 7 months up to 10 years after stroke (Table 2). Verbal fluency and executive function also remained stable between 1-week to 10-year follow-ups (Table 2) when tested with D-KEFS-Verbal fluency (18) and TMT-B (19, 20).

### Dynamic Process Speed

Process speed assessed with a WAIS-Symbol search demonstrated a significant decrease (6.33, 95%CI— 3.65 to 9.02, *p* = 0.03) after 10 years compared to the 7-month follow-up. However, WAIS-Coding, not TMT-A, demonstrated a significant speed enhancement at sub-acute phase but returned to the former level at the chronic stage (Table 2).

### Improvement in Global Cognition

The global cognition at 10-year follow-up assessed by MMSE showed a median score of 29 with 25–75% percentile (27–30). Compared to the results at 7 months after stroke onset, with a median score of 27 with 25–75% percentile (25–29), there was a weak but significant improvement in global cognition (p = 0.02) among participants (Table 2).

### Correlation Between Risk Factors and Cognition

**Figure 3:**
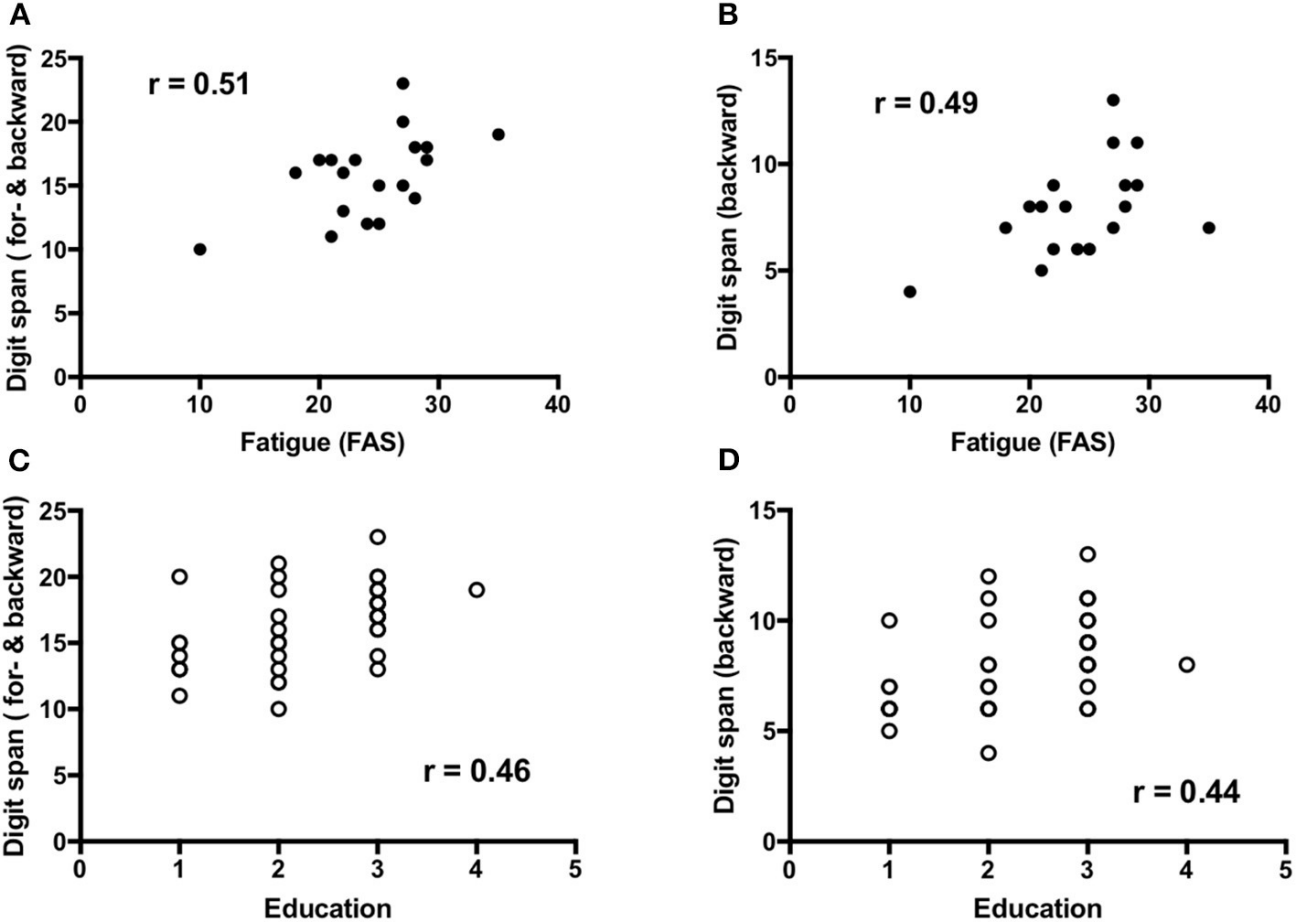
Correlations between working memory and fatigue **(A,B)** as well as education **(C,D)**.

Fatigue (*n* = 19) demonstrated a low to moderate but significant positive correlation to working memory that is forward and backward digit span [*r* = 0.51, 95%CI (0.06–0.79), *p* = 0.03] and backward digit span [*r* = 0.49, 95%CI (0.03–0.78), *P* = 0.03] (Figures 3A,B), and visuospatial function [*r* = 0.48, 95%CI (0.01–0.77), *p* = 0.04]. Education (*n* = 37) showed a low but significant positive correlation with working memory, that is forward and backward digit span [*r* = 0.46, 95%CI (0.15–0.69), *p* = 0.004] and backward digit span [*r* = 0.44, 95%CI (0.12–0.67), *p* = 0.007] (Figures 3C,D) as well as forward digit span [*r* = 0.38, 95%CI (0.05–0.63), *p* = 0.02].

**Figure 4:**
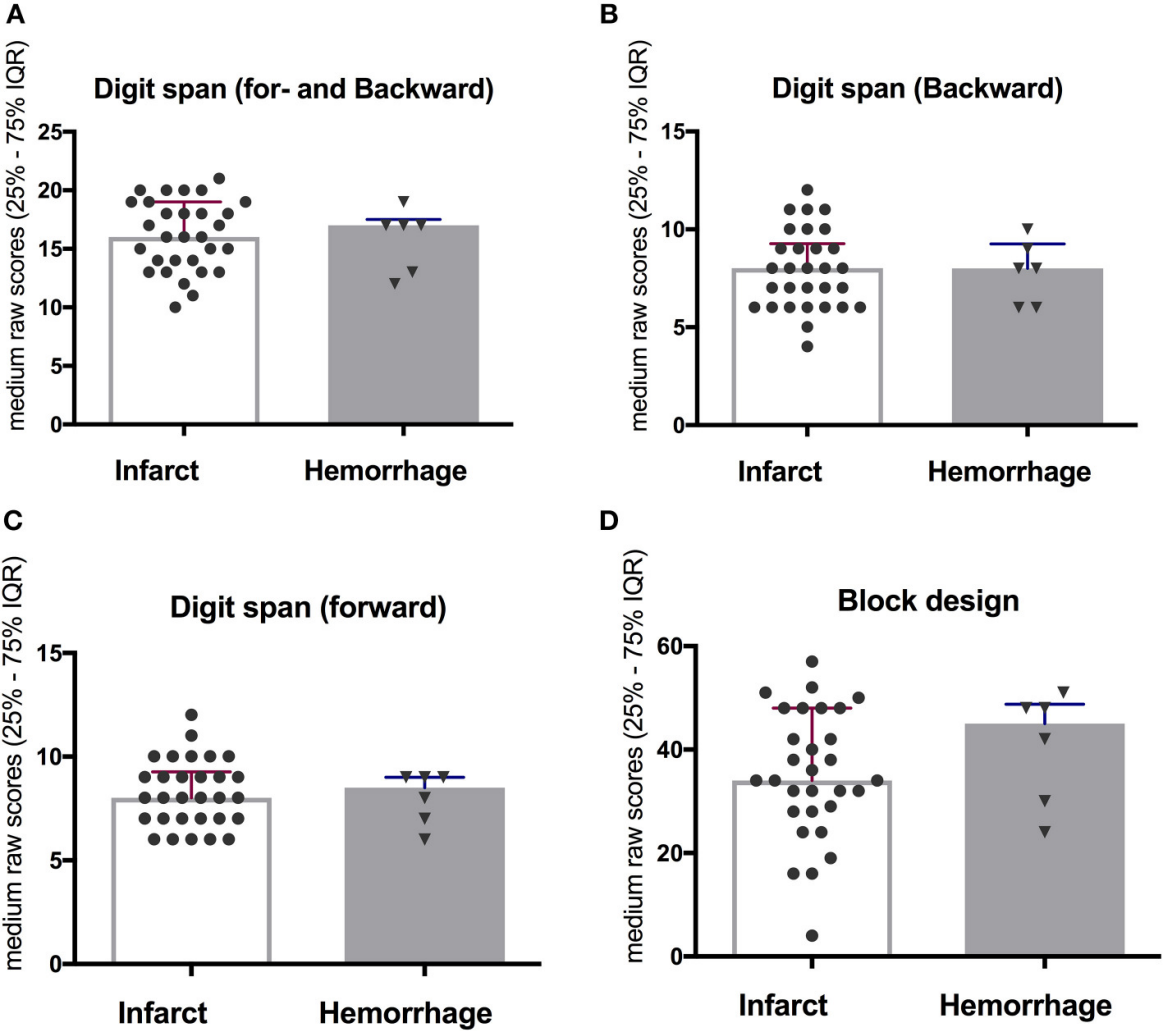
No significant difference when comparing between ischemic and hemorrhagic stroke on working memory **(A–C)** and visuospatial function **(D)** at 10-year follow-up.

No significant differences were observed in the working memory and visuospatial function when comparing different stroke sub-type (ischemic vs. hemorrhagic infarct) at 10-year follow-up (Figure 4). Notably, only six participants suffered hemorrhagic infarct in the cohort.

There were no correlations demonstrated between cognitive improvements and age, gender, anxiety, or depression among participants at 10-year follow-up.

## Discussion

The current study provides a unique longitudinal cognitive evaluation of young stroke survivors using a comprehensive neuropsychological test battery repetitively from acute (1-week), subacute (7-month) to chronic (10-year) stages after stroke onset. To our knowledge, no such valuable cognitive data have been reported previously. We found significant improvements and recoveries with respect to working memory, visuospatial function, and global cognition, after initial post-stroke cognitive decline among the participants at 10 years after stroke. Visuospatial function improved already at 7 months post-stroke but working memory was enhanced mainly at the chronic phase, 10 years after stroke. The remaining cognitive domains, such as attention, immediate and delayed memory, and verbal and executive function, remained relatively stable over 10 years after initial post-stroke declines. Processing speed was the only domain in the cohort that demonstrated a significant decrease in one of three subtests at 10-year follow-up compared to 7-month follow-up. Education level and fatigue, but not stroke sub-type, age, and gender, showed low to moderate positive correlations with cognitive improvements among participants.

The most striking finding in the current study was significant improvement of working memory in all Digit span assessments at 10 years after stroke. No such late cognitive recovery among stroke survivors has previously been reported according to our knowledge, although good functional outcome is common among young stroke survivors (21). This surprising result is truly encouraging because working memory plays an important role in everyday functions. The amelioration of working memory capacity among the participants could implicate a potential enhancement of other complex cognitive functions (22), such as reading comprehension, learning ability, and executive function (9) in their daily lives. This role is in fact supported by basic characteristic data in the present cohort, where more than 90% of participants were independent in their daily activities (mRS = 0–2) and one fourth of participants remained working part or full time at 10 years after stroke. In fact, improvement of working memory in the present cohort reached a similar level to their non-stroke peers (14), suggesting that a true recovery of working memory does occur among these young stroke survivors.

Our results are however contradictory to two previous studies with relatively large samples (515 and 277 stroke survivors, respectively) showing cognitive decline at 6 or 10 years after stroke (3, 4). The weaknesses of these previous studies are their cognitive assessments at only a single time-point, meaning that they were unable to detect dynamic cognitive improvements over time after stroke. Compared to the Digit span we used to examine working memory, Schaapsmeerder et al. employed the paper and pencil memory scanning test (PPMST) to assess working memory (3) and no specific working memory was assessed in another previous study (4). The discrepancy of these two assessment tools and characteristics of different samples may have contributed to the differences in the results. In addition, a relatively higher education level (almost half of the participants had a university degree) in our cohort could also partially explain the improvements of cognitive outcomes at the 10-year post-stroke, due to the fact that we and others (14, 23) found strong evidence that education plays a significant positive role in working memory.

Intriguingly, Digit span forward (which considers pure short-term memory and rote learning) had improved already at sub-acute phase after stroke; but the more challenging Digit span backward and Digit span total score reflected real working memory recovered later at the chronic stage. This result is highly encouraging because it indicates that a delayed improvement of working memory does occur at least among these younger community-dwelling stroke survivors at chronic phase. This finding suggests that more complicated functions, such as working memory, may require longer time (more than a year/ years) to recover. This prolonged improvement of working memory after stroke illustrated in this study provides important implications for cognitive rehabilitation, driving, and care planning in clinical practice.

Compared to working memory, visuospatial function assessed with Block design demonstrated another temporal profile. There was an early significant recovery to a similar level as their peers (14) already at sub-acute phase and it remained stable at 10-year follow-up. Although this result is in line with previous studies (10, 24), the different recovery trajectories in varying domains are intriguing. Notably, no significant alterations were observed over time in RCFT-copy test as well as Bell-test, results which could be explained by a ceiling effect on these tests already at the acute stage. This result may indicate a certain difficulty of NPA required at the acute phase in order to detect the improvement over time.

Beside long-term recoveries on working memory and visuospatial function, our results demonstrated that other cognitive domains such as verbal fluency, immediate and delayed memory, and executive function were relatively stable over 10 years. This is important knowledge for clinicians and patients because a significant amount of previous data could only point out the cognitive decline after stroke at certain time-points (3, 4). Our current results suggest no further deterioration in overall cognition among these young stroke survivors over 10 years' time, despite the initial declines after stroke. This result may imply that our participants had in fact slightly improved their cognition to compensate for age-related declines. Our results provide important information to many young stroke survivors and clinicians because they provide evidence counteracting the previous assumption regarding stroke survivors acquiring a faster rate of cognitive decline over the years (4), at least among younger stroke survivors. As stated previously, different assessments and sample characteristics, such as age and education, may explain some discrepancies between the studies.

The reason for the cognitive improvements in the current study was not investigated at this stage. Because no organized cognitive rehabilitation was supplied after the initial hospitalization in this cohort, participants' struggle to be active and/or live independently in their daily lives may presumably be a crucial element for their cognitive improvements. These daily activities could possibly trigger activity-induced neuroplasticity (25) to further ameliorate cognitive recovery. Furthermore, a better collateral blood flow with a more pronounced neuronal plasticity as well as absence/less of neurodegenerative pathology may facilitate cognitive enhancement in young stroke survivors (21, 26). Therefore, active participation in daily activity should be taken into account when cognitive rehabilitation is planned in clinical settings.

In contrast, process speed examined by a Symbol search showed significant decline at 10-year follow-up but not process speed measured with Coding and TMT-A. A possible explanation could be that Coding demands cognitive processes other than Symbol search, such as visual short-term memory, visuo-motor coordination, and attention (14). The pure decline of processing speed may be compensated by other cognitive improvements mentioned above. The decline of processing speed on Symbol search may be an expected age-related change (27) when the participants aged, with a mean age of 54–64 years during 10-year follow-up in the current study. Beside stroke, age alone has important impact on cognitive performance due to structural changes in the aged brain (27, 28).

Global cognition measured by MMSE also improved by on average two points between 7-month and 10-year follow-ups in the present study. Together with previous studies showing global cognitive recovery from 3 weeks to 1 year after stroke onset (8, 9, 11, 12), the current results suggest that cognitive recovery may begin early after initial cognitive decline, and continue to improve over a long period of time (i.e., more than a year or years after stroke). Improvements of global cognition are supported by neuropsychological data because most cognitive domains measured by the neuropsychological test battery showed either marked improvements or stable results over time. The concordance between global and domain-specific cognitions suggest strongly that stroke survivors may improve their cognitive outcome over a long time at a young age.

A small but significant positive association was found between level of education and working memory. This result is in line with early evidence that working memory capacity is correlated with learning outcomes in literacy and numeracy (14, 29). These education effects may be also mediated by age (23). However, different stroke sub-type did not influence cognitive recovery in our cohort with notably very limited sample size. Furthermore, stroke localization could play a very important role in cognitive recovery. Unfortunately, the clinical characteristics in the cohort were collected from the Riksstroke registry, where stroke localization was not registered.

We are surprised about the positive association between fatigue and improved cognitive function. This relation could possibly be explained by the fact that fatigue was assessed by answering questionnaires at home prior to NPA, and thus mainly reflected fatigue level in participants' daily lives. Presumably, those participants who performed well in the NPA and struggle in daily life have to use more energy to achieve better functional outcomes with fatigue as a drawback. This presumption may suggest various reasons behind the fatigue at different stages after stroke. Therefore, awareness of the importance of balance between activity and rest over time should be enhanced in both stroke survivors and medical staff.

The current study has several unique strengths. We demonstrated repeatedly domain-specific cognitive outcomes by NPAs at acute, sub-acute, and chronic phases over 10 years after stroke onset, providing us with a distinctive opportunity to detect longitudinal cognitive alterations over time. Moreover, trajectory of post-stroke cognition was examined by both MMSE and NPAs in the same cohort. It provides not only more comparative information between global- and domain-specific cognition but also more comprehensive information regarding multiple cognitive domains: process speed, visual attention, as well as visuospatial, executive, and verbal functions, and in addition working, immediate, and delayed memory types. Furthermore, all assessors were blind to the previous assessments in order to reduce possible sources of bias.

However, some methodological limitations need to be addressed. First, we are aware of the small number of participants and some variations of the number of participants at early time-points in the current study. The current findings need to be replicated in other studies with a larger sample size. Second, no cognitive data were collected for those who denied participating in the study. One may assume that the deniers may have worse clinical outcomes. However, demographic characteristics did not show any significant differences between participants and deniers, indicating that selection bias was unlikely during the follow-ups. Third, our results were generalized only from community-dwelling participants without aphasia. Nevertheless, rather than generalizing the findings to the entire young stroke population, the current study suggests mainly that cognitive recovery does occur during both sub-acute and chronic phases after first-ever stroke, at least among some young stroke survivors who are living in the community.

This study demonstrated that significant cognitive recoveries on visuospatial function and working memory do occur at sub-acute and chronic stages after initial post-stroke cognitive decline in young community-dwelling adults after first-ever stroke. No further deterioration in overall cognition occurred among these young stroke survivors over 10 years' time after stroke. The age-related decline of processing speed at 10-year follow-up could be compensated by other cognitive improvements and stabilizations over time. This finding fills a gap of knowledge concerning the dynamic trajectory of post-stroke cognition over 10 years after stroke. The current results may supply strong clinical implications on rehabilitation and care planning in clinical practice.

## Author Contributions

EE contributed to study conception, sample collection, interpretation of data, and revising the manuscript, provided final approval of the version to be published; agreed to be accountable for all aspects of the work. XH contributed to study conception, supervision of the acquisition, interpretation of data, drafting, and revised the manuscript including the figures, tables, and references and provided final approval of the version to be published and agreed to be accountable for all aspects of the work.

### Conflict of Interest Statement

The authors declare that the research was conducted in the absence of any commercial or financial relationships that could be construed as a potential conflict of interest.

## Abbreviations

NPA: Neuropsychological assessment
WAIS-IV: Wechsler Adult Intelligence Scale-IV
RCFT: Rey complex figure test and recognition trial
CD: Claeson-Dahl
FAS: a part of the test battery
Delis-Kaplan Executive Function System (D-KEFS): named letter fluency
MMSE: Mini mental state examination
mRS: Modified Rankin Scale.
